# Supplementary material for: Facile Solvent-Free Synthesis of Metal Thiophosphates and Their Examination as Hydrogen Evolution Electrocatalysts
Source: Molecules. 2022 Aug 9;27(16):5053. doi: 10.3390/molecules27165053 (PMC9413033; doi:10.3390/molecules27165053)
Supplement: Supplementary file 1 [file molecules-27-05053-s001.zip › molecules-1815512-supplementary.pdf]

Supplementary Information for

Facile Solvent-Free Synthesis of Metal Thiophosphates and Their Examination as Hydrogen Evolution Electrocatalysts

Nathaniel Coleman Jr., Ishanka A. Liyanage, Matthew D. Lovander, Johna Leddy, Edward G. Gillan*

Department of Chemistry, University of Iowa, Iowa City, Iowa 52242 USA
Email: edward-gillan@uiowa.edu

Table of Contents for Supporting Information (listed in order they appear in the text)

Table S1 - Structural information for M-P-S structures.

Figure S1 - FT-IR data on M-P-S products.

Table S2 - UV-vis DRS summary results

Table S3 - Magnetic susceptibility results

Table S4 - Thermochemical data and results comparing MP_x and MS_x reactions

Figure S2 – Representative MPS_3 LSV curves with and without 85% iR compensation.

Figure S3A - LSV overlay HER results for $CoPS_3$ (uncompensated)

Figure S3B - LSV overlay HER results for $CoPS_3$ (85% iR compensation)

Figure S4A - LSV overlay HER results for $NiPS_3$ (uncompensated)

Figure S4B - LSV overlay HER results for $NiPS_3$ (85% iR compensation)

Figure S5A - LSV overlay HER results for $FePS_3$ (uncompensated)

Figure S5B - LSV overlay HER results for $FePS_3$ (85% iR compensation)

Figure S6 – Representative Tafel plots for $CoPS_3$, $NiPS_3$, and Pt/C powders.

Figure S7 – Representative ECSA analysis of current versus scan rate results.

Figure S8 - 18-hour constant potential chronoamperometry (CA) HER experiments for MPS_3 catalysts using a platinum counter electrode (CE).

Figure S9 - 18-hour constant potential chronoamperometry (CA) HER experiments for MPS_3 catalysts using a graphite CE.

Figure S10A – Microprobe analysis of $NiPS_3$ on C_{wax} tip after 18-hour CA using Pt CE.

Figure S10B – Microprobe analysis of $FePS_3$ on C_{wax} tip after 18-hour CA using Pt CE.

Figure S11 – SEM images of MPS_3 materials embedded on C_{wax} after 18-hour CA HER experiments.

Figure S12A – EDS elemental maps for $FePS_3$ particles on C_{wax} tips after CA experiments.

Figure S12B – EDS elemental maps for $CoPS_3$ particles on C_{wax} tips after CA experiments.

Figure S12C – EDS elemental maps for $NiPS_3$ particles on C_{wax} tips after CA experiments.

Figure S13 – Images of electrochemical cell, C_{wax} tip connections, and use in XRD analysis.

Table S1. M-P-S crystal structure information

Compound	Unit Cell & Space Group (number)	Lattice Parameters	Cell Volume (Å^3)	Ref.
$FePS_3$	Monoclinic C2/m (12)	a = 5.95 Å; b = 10.30Å c = 6.72Å; β = 107.16°	393.43	[1]
$CoPS_3$	Monoclinic C2/m (12)	a = 5.90 Å; b = 10.22Å c = 6.66 Å; β = 107.17°	383.71	[1]
$NiPS_3$	Monoclinic C2/m (12)	a = 5.81 Å; b = 10.07 Å c = 6.63 Å; β = 106.98°	371.23	[2]
Cu_3PS_4	Orthorhombic Pmn21 (31)	a = 7.28 Å; b = 6.34 Å c = 6.08 Å	280.43	[3]

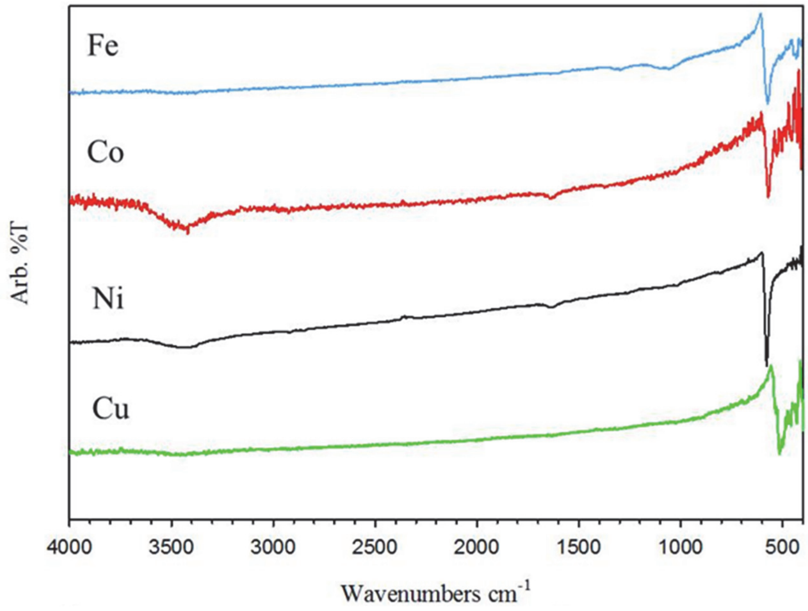

Figure S1. FT-IR data on KBr pressed pellets containing M-P-S samples synthesized from MCl_x and P+S reaction products. Broad adsorbed water peaks on KBr are at ~3400 cm^{-1} and ~1600 cm^{-1}. The black colored MPS_3 samples absorb across the entire spectrum. The MPS_3 samples have an intense peak at 570 - 580 cm^{-1} region consistent with P-S stretching vibrations.

Table S2. Summary of estimated M-P-S band gaps calculated from solid-state DRS absorption data.

**Products**	**Onset Abs. (nm)**	**Est. Band Gap (eV)**	**Literature Band Gap (eV)**
**$FePS_3$ (P+S)**	760	1.63	1.50 [4]
**$FePS_3$ (P_2S_5 + P)**	780	1.59	1.50 [4]
**$CoPS_3$ (P+S) (contains $CoP_{0.5}S_{1.5}$)**	940	1.32	1.40 [5-6]
**$CoPS_3$ (P_2S_5 + P)**	745	1.66	1.40 [5-6]
**$NiPS_3$ (P+S) (contains NiS_2)**	915	1.36	1.60 [4]
**$NiPS_3$ (P_2S_5 + P)**	740	1.68	1.60 [4]
**Cu_3PS_4 (P+S)**	520	2.38	2.36 [7]
**Cu_3PS_4 (P_2S_5 + P)**	525	2.36	2.36 [7]

Table S3. Magnetic data for M-P-S samples. The spin only cases assume high spin M^{2+} for MPS_3.

**Products**	**M^{n+}, d^n, spin-only μ_B (BM)**	**Lit. values μ_B (BM) [8]**	**Experimental χ_M (x 10^{-3}) cm^3/mol**	**Experimental μ_B (BM)[1]**
**$FePS_3$ (P+S)**	Fe^{2+}, d^6, 4.90	4.94	10.3	4.98
**$FePS_3$ (P_2S_5 + P)**	Fe^{2+}, d^6, 4.90	4.94	11.7	5.28
**$CoPS_3$ (P+S) (contains $CoP_{0.5}S_{1.5}$)**	Co^{2+}, d^7, 3.87	4.93	3.34	2.87
**$CoPS_3$ (P_2S_5 + P)**	Co^{2+}, d^7, 3.87	4.93	5.87	3.02
**$NiPS_3$ (P+S) (contains NiS_2)**	Ni^{2+}, d^8, 2.83	3.90	1.79	2.10
**$NiPS_3$ (P_2S_5 + P)**	Ni^{2+}, d^8, 2.83	3.90	2.00	2.23
**Cu_3PS_4 (P+S)**	Cu^{+}, d^{10}, 0.00	diamagnetic	-0.076	0.46
**Cu_3PS_4 (P_2S_5 + P)**	Cu^{+}, d^{10}, 0.00	diamagnetic	-0.020	0.48

1) magnetic moment calculated after diamagnetic corrections to measured molar susceptibility.

Table S4. Comparison of thermochemical and reaction product info for phosphorus and sulfur reactions with metal halides.[9-10] Reactions were run in evacuated ampoules to 500 °C using stoichiometrically balanced reactions to produce PCl_3 or S_2Cl_2 byproducts.

Reaction	Target Product	ΔH_{rxn} (kJ/mol)	XRD results
$FeCl_3$ + P	FeP_2	-113	FeP_2
$CoCl_2$ + P	CoP_3	-83	CoP_3
$NiCl_2$ + P	NiP_2	-19	NiP_2
$CuCl_2$ + P	CuP_2	-95	CuP_2
$FeCl_3$ + S	FeS_2	+189	**NR,** $FeCl_2$-H_2O
$CoCl_2$ + S	Co_2S_3	+296	**NR,** $CoCl_2$-$6H_2O$
$NiCl_2$ + S	NiS_2	+158	**NR,** $NiCl_2$
$CuCl_2$ + S	CuS	+145	**NR,** CuCl

NR = no detectable reaction

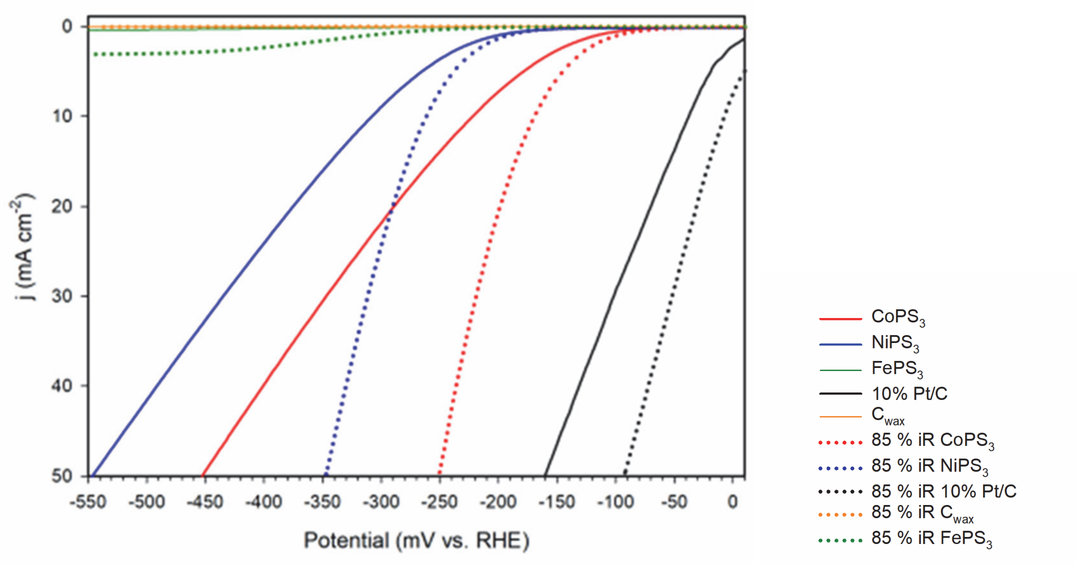

Figure S2. Overlay graph of representative LSV data for MPS_3, Pt/C, and C_{wax} in 0.5 M H_2SO_4 with and without 85% iR compensation. Curves with dashed lines have 85% iR instrument correction applied.

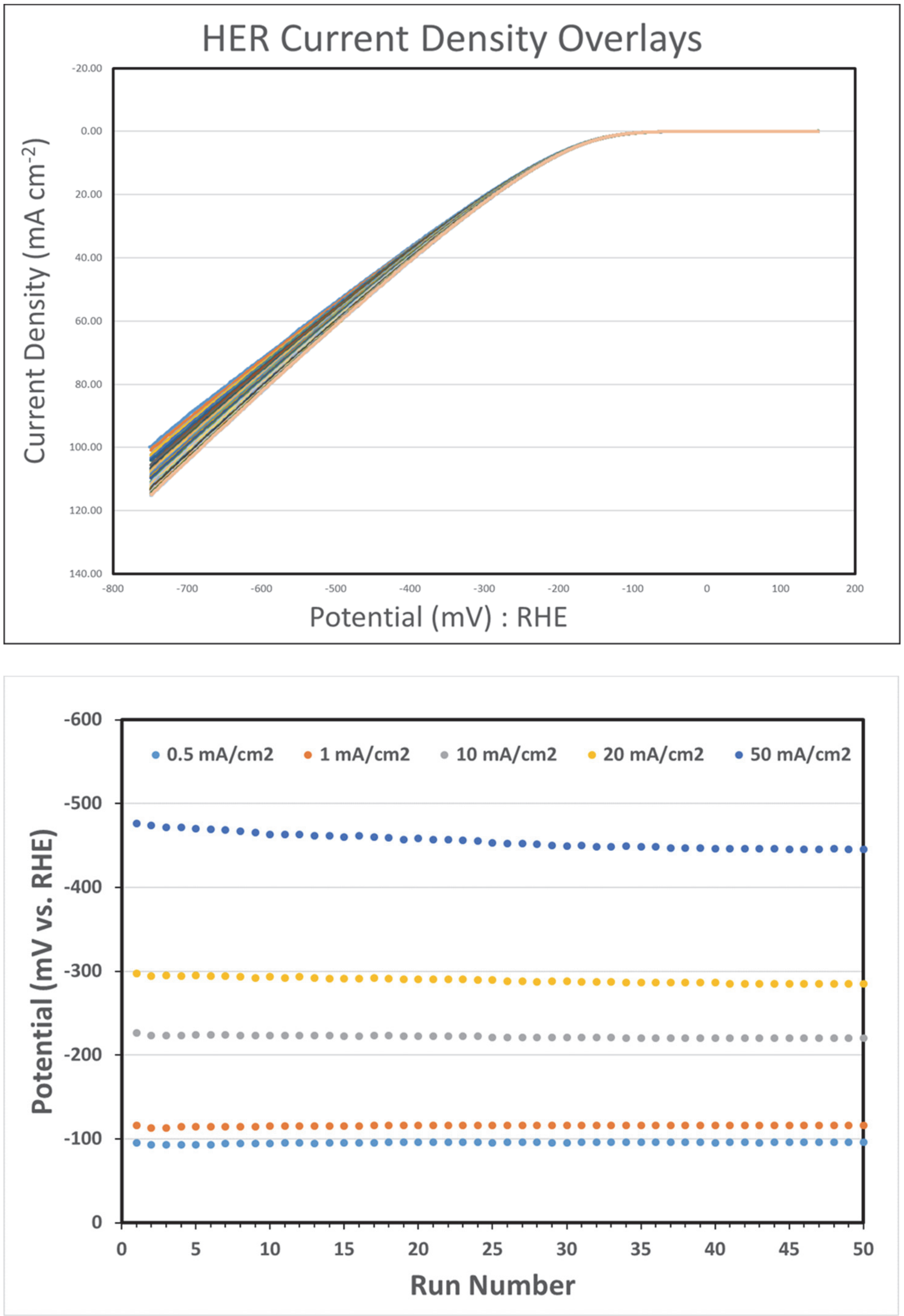

Figure S3A. Overlay LSV graphs (top) for 50 runs using $CoPS_3$ catalyst in 0.5 M H_2SO_4 (5 mV/s scan rate, graphite counter, no iR compensation). Bottom plot shows applied potentials required to achieve specific mA/cm^2 values in each LSV run that is in the LSV overlay plot.

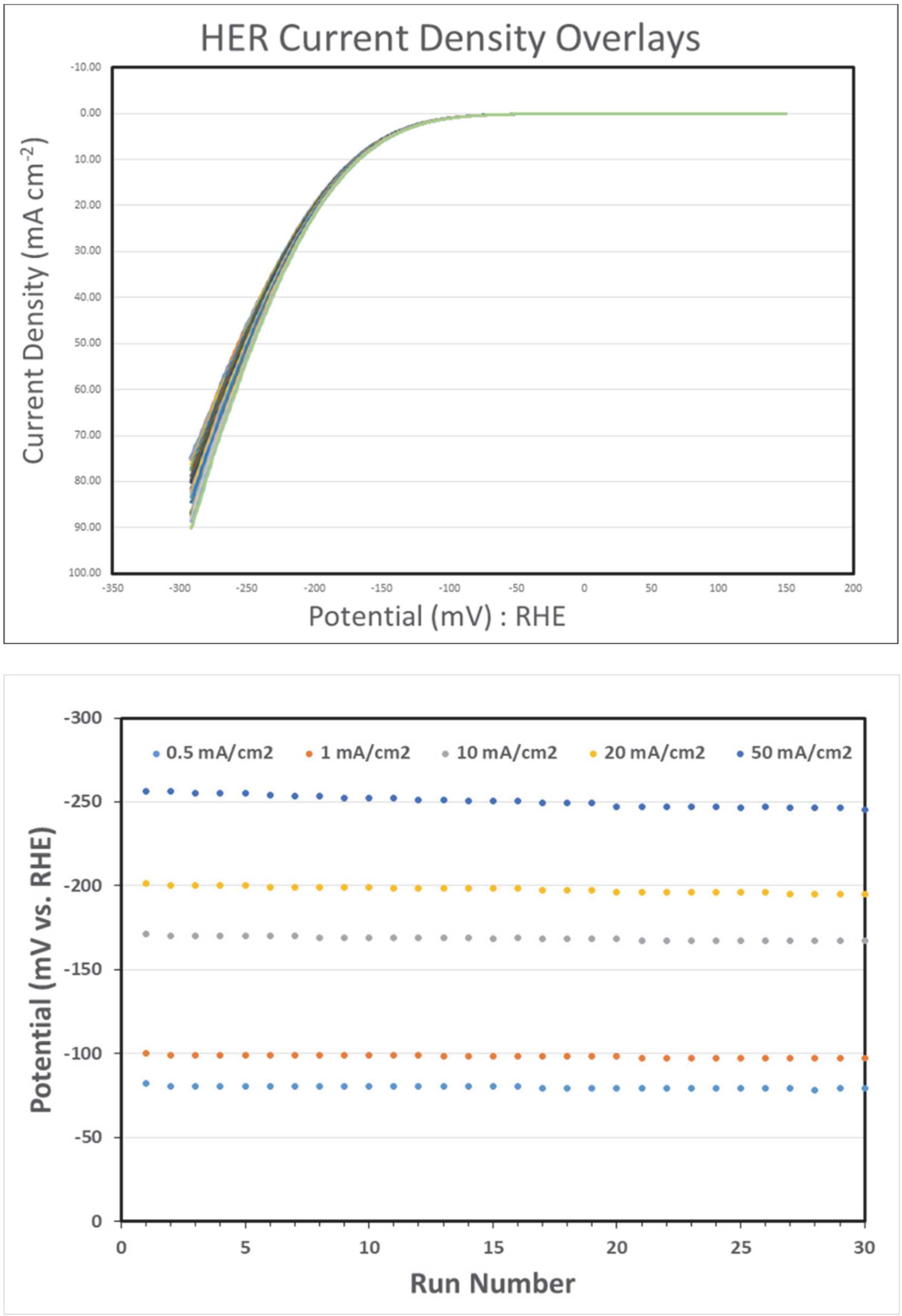

Figure S3B. Overlay LSV graphs (top) for 30 runs using $CoPS_3$ catalyst in 0.5 M H_2SO_4 (5 mV/s scan rate, graphite counter, 85% iR compensation, Rcell = 64 Ω). Bottom plot shows applied potentials required to achieve specific mA/cm^2 values in each LSV run that is in the LSV overlay plot.

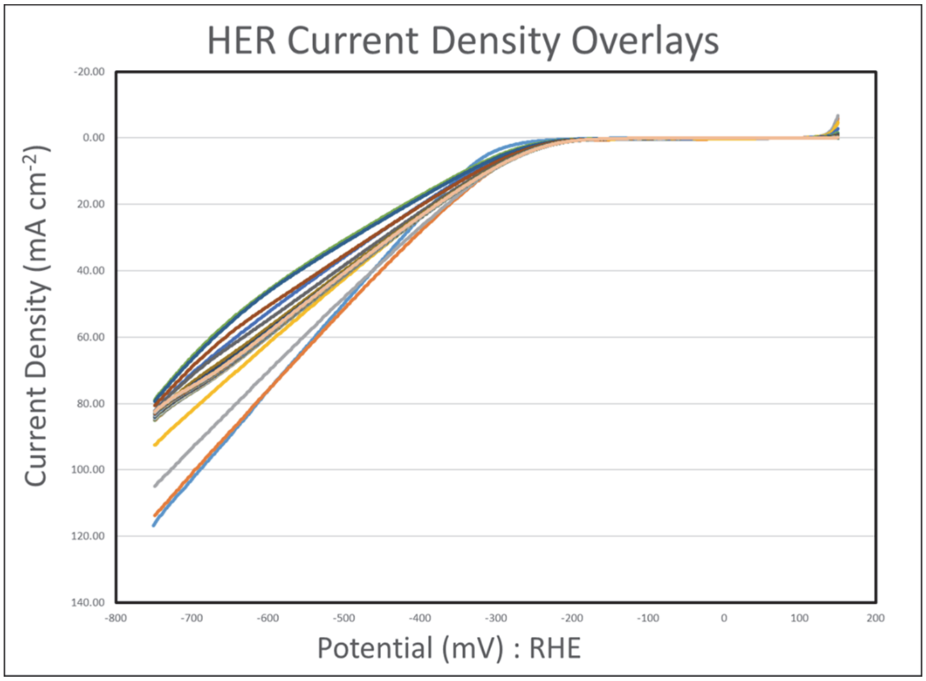

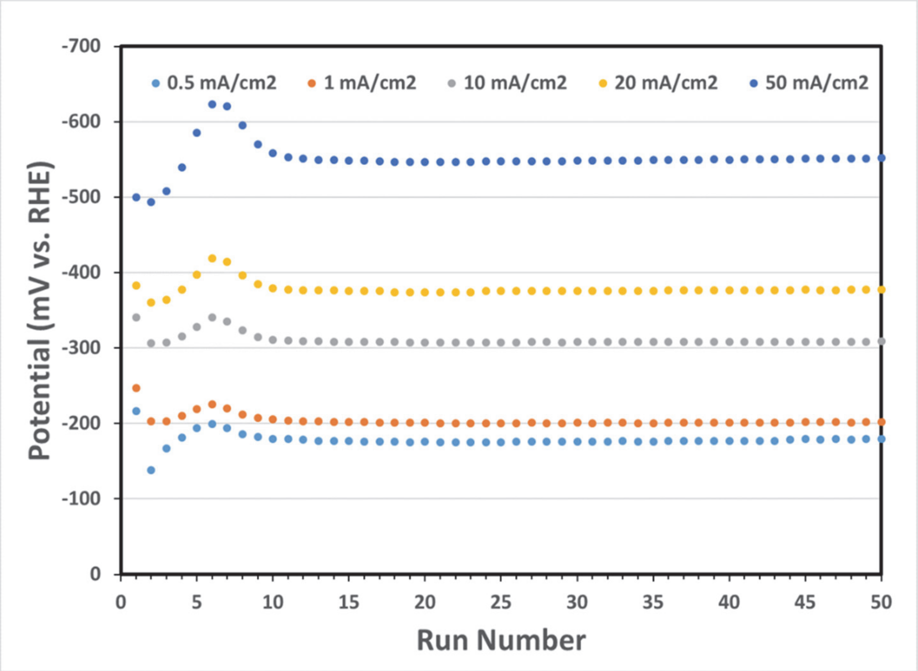

Figure S4A. Overlay LSV graphs (top) for 50 runs using $NiPS_3$ catalyst in 0.5 M H_2SO_4 (5 mV/s scan rate, graphite counter, no iR compensation). Bottom plot shows applied potentials required to achieve specific mA/cm^2 values in each LSV run that is in the LSV overlay plot.

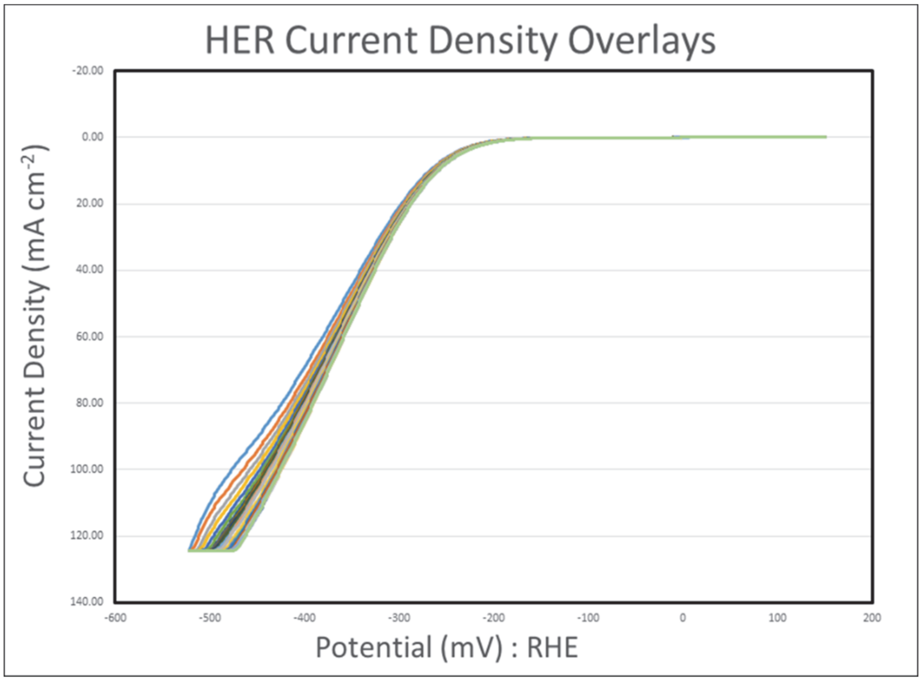

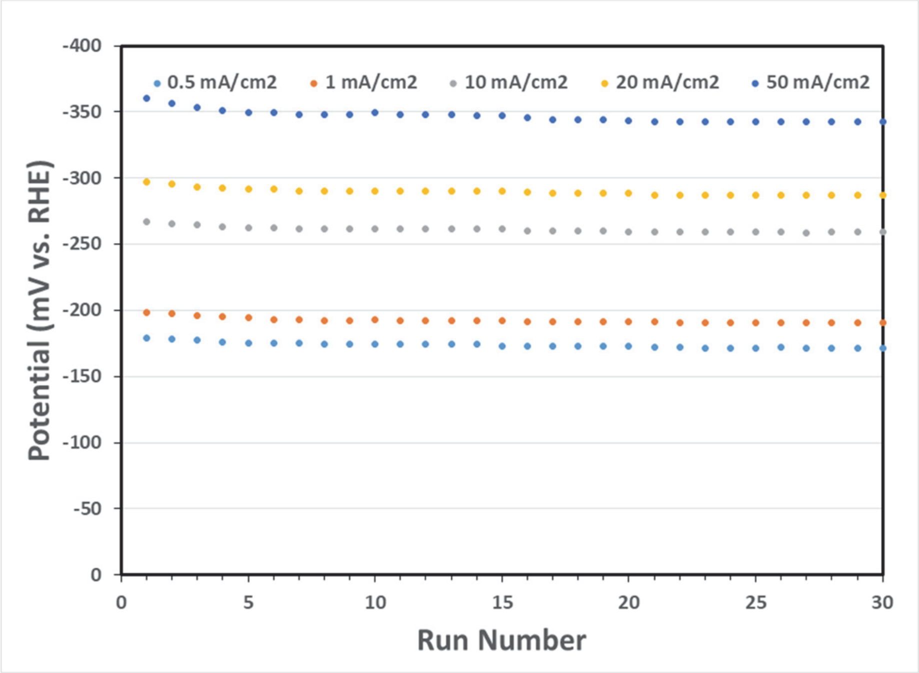

Figure S4B. Overlay LSV graphs (top) for 30 runs using $NiPS_3$ catalyst in 0.5 M H_2SO_4 (5 mV/s scan rate, graphite counter, 85% iR compensation, Rcell = 68 Ω). Bottom plot shows applied potentials required to achieve specific mA/cm^2 values in each LSV run that is in the LSV overlay plot.

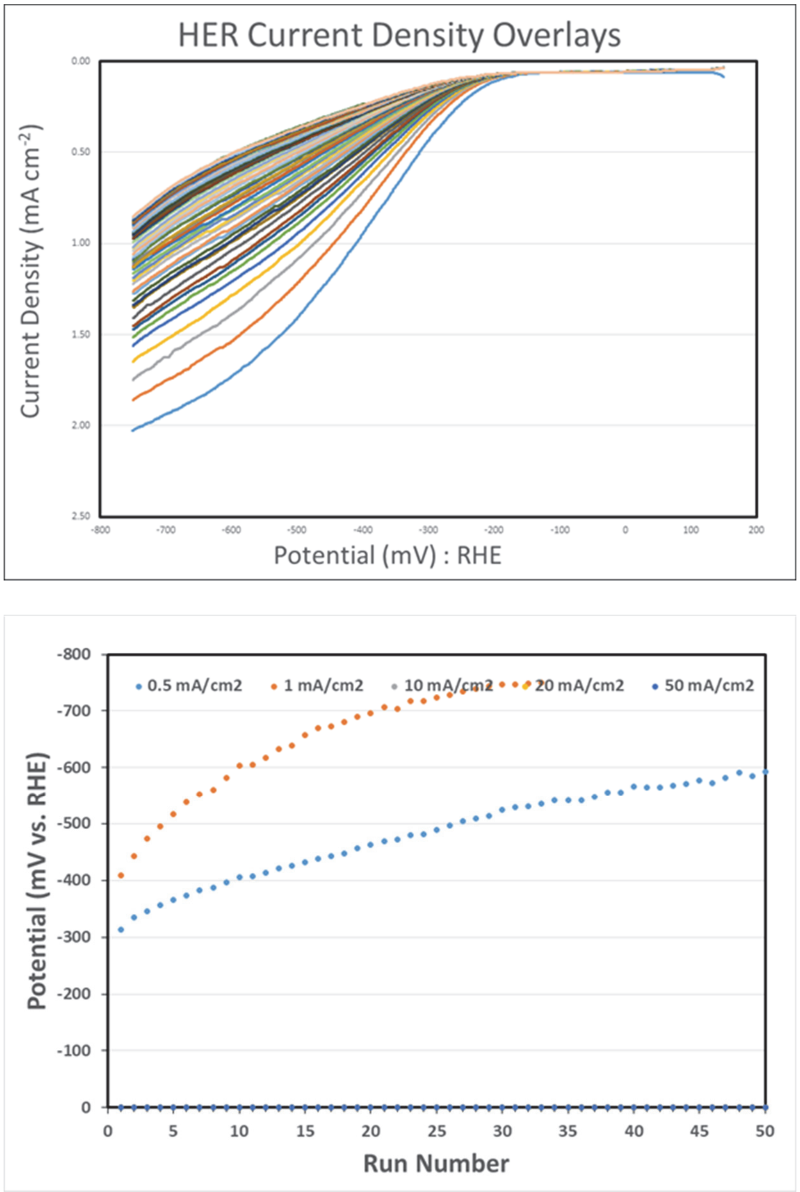

Figure S5A. Overlay LSV graphs (top) for 50 runs using $FePS_3$ catalyst in 0.5 M H_2SO_4 (5 mV/s scan rate, graphite counter, no iR compensation). Bottom plot shows applied potentials required to achieve specific mA/cm^2 values in each LSV run that is in the LSV overlay plot.

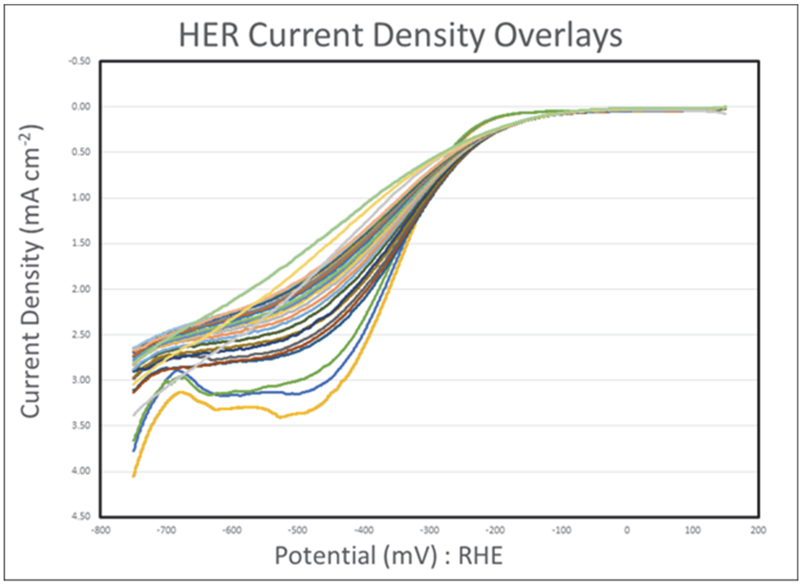

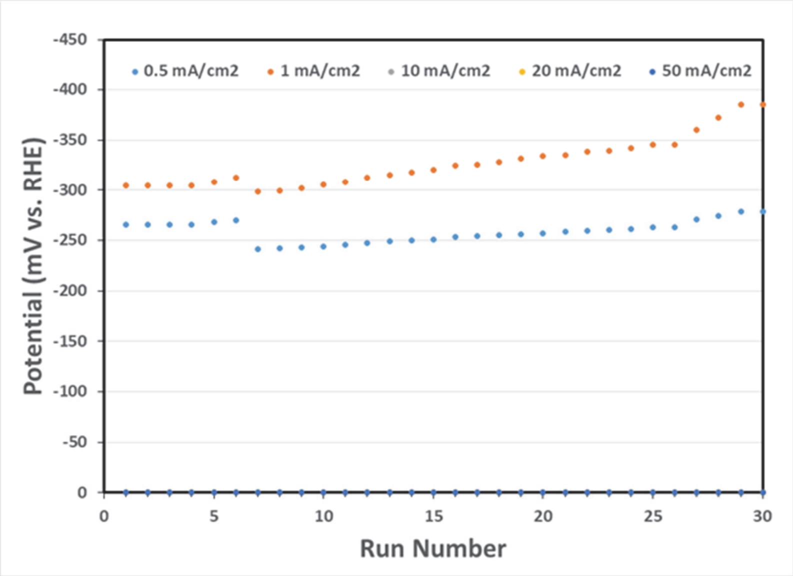

Figure S5B. Overlay LSV graphs (top) for 30 runs using $FePS_3$ catalyst in 0.5 M H_2SO_4 (5 mV/s scan rate, graphite counter, 85% iR compensation, Rcell = 350 Ω). Bottom plot shows applied potentials required to achieve specific mA/cm^2 values in each LSV run that is in the LSV overlay plot.

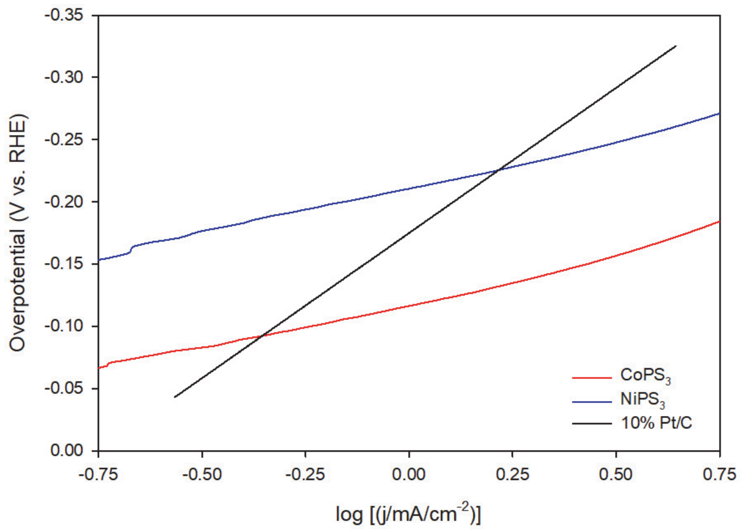

Figure S6. Representative Tafel graphs for $CoPS_3$, $NiPS_3$, and Pt/C powders. Data from 50 LSV runs overlay well with these graphs and deviations from the mean are reported in Table 3 of the main paper. Linear regression equations for these data are: $CoPS_3$ (y = -0.071x – 0.1173, R^2 = 0.9971), $NiPS_3$ (y = -0.0858x - 0.2006, R^2 = 0.9987), and 10% Pt/C (y = -0.0492x + 0.0202, R2 = 0.9983)

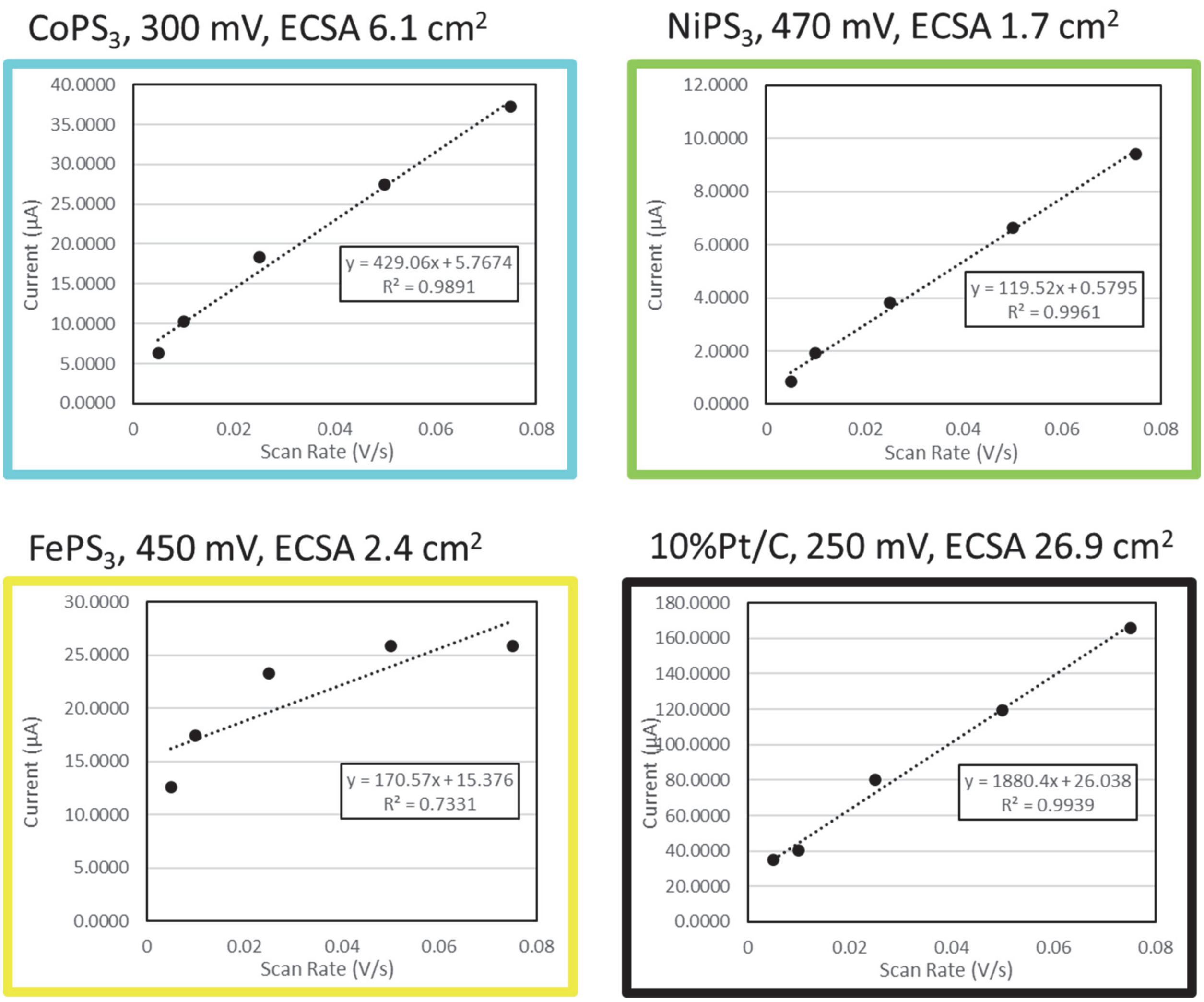

Figure S7. Analysis of scan rate data from CV runs used to calculate ECSA values in Table 3.

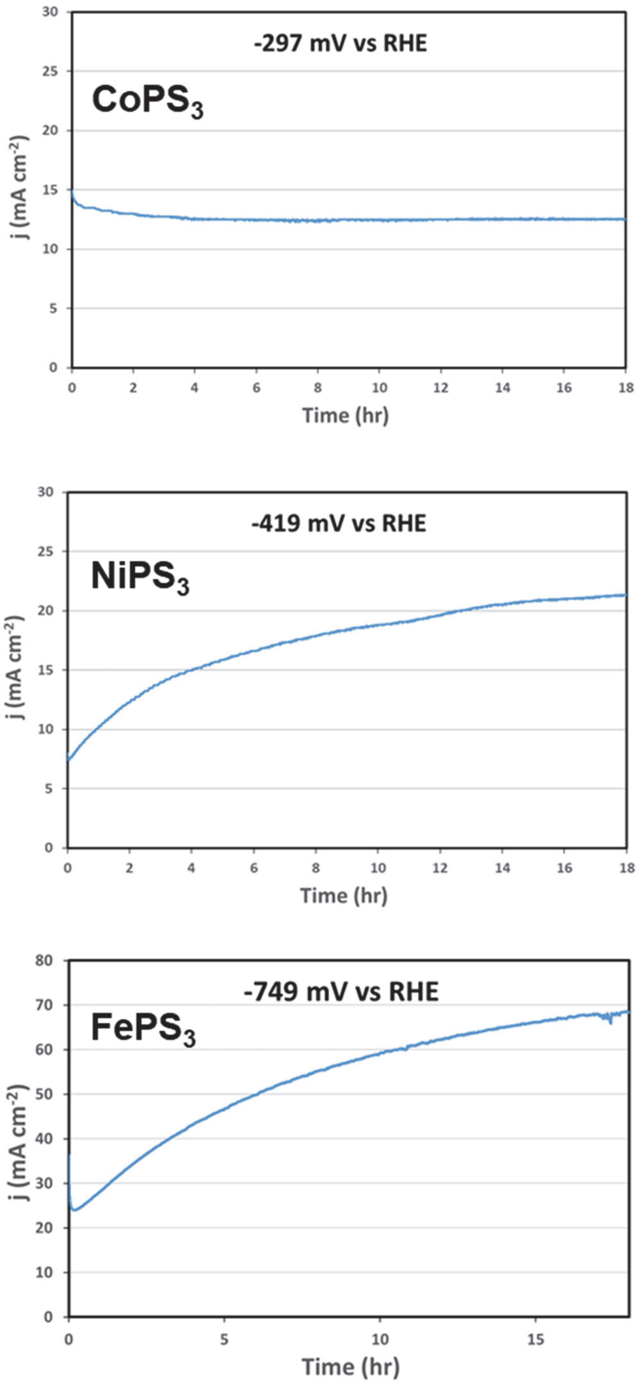

Figure S8. 18-hr chronoamperometry (CA) HER data for MPS_3 (0.5 M H_2SO_4, platinum counter, no iR compensation): $CoPS_3$ top, $NiPS_3$ middle, $FePS_3$ bottom. Constant applied potential values for experiment is listed in each graph.

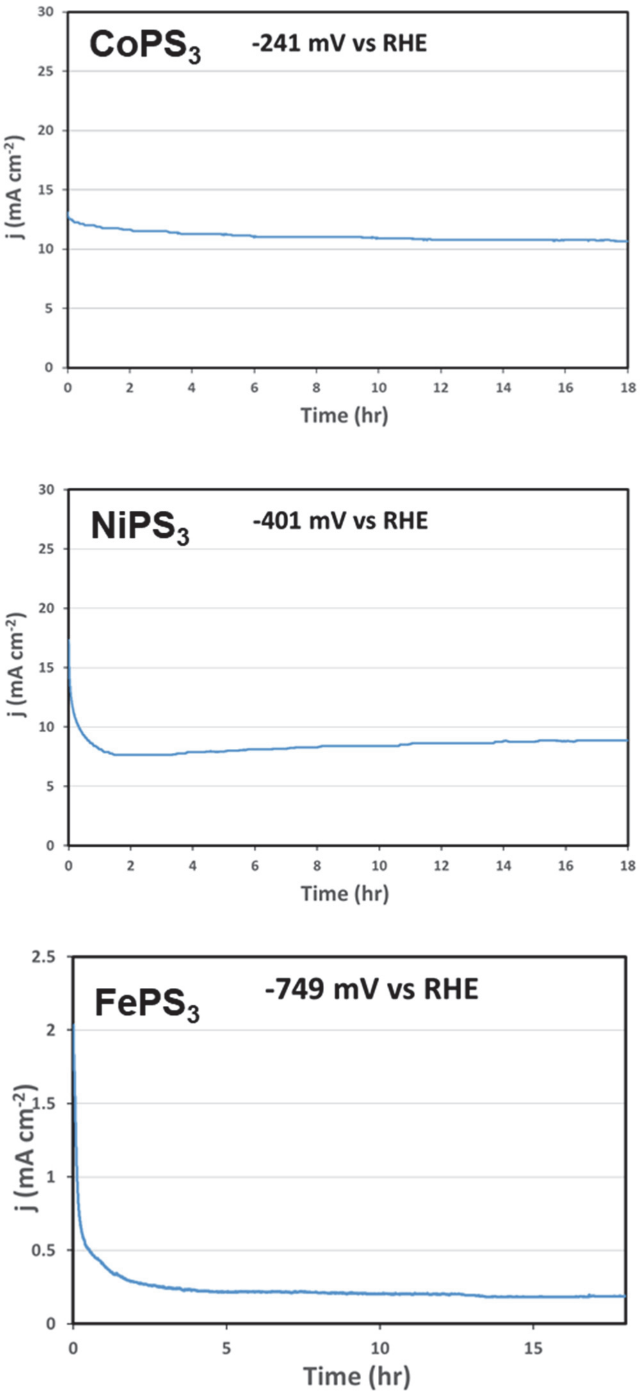

Figure S9. 18-hr chronoamperometry (CA) HER data for MPS_3 (0.5 M H_2SO_4, graphite counter, no iR compensation): $CoPS_3$ top, $NiPS_3$ middle, $FePS_3$ bottom. Constant applied potential values for experiment is listed in each graph.

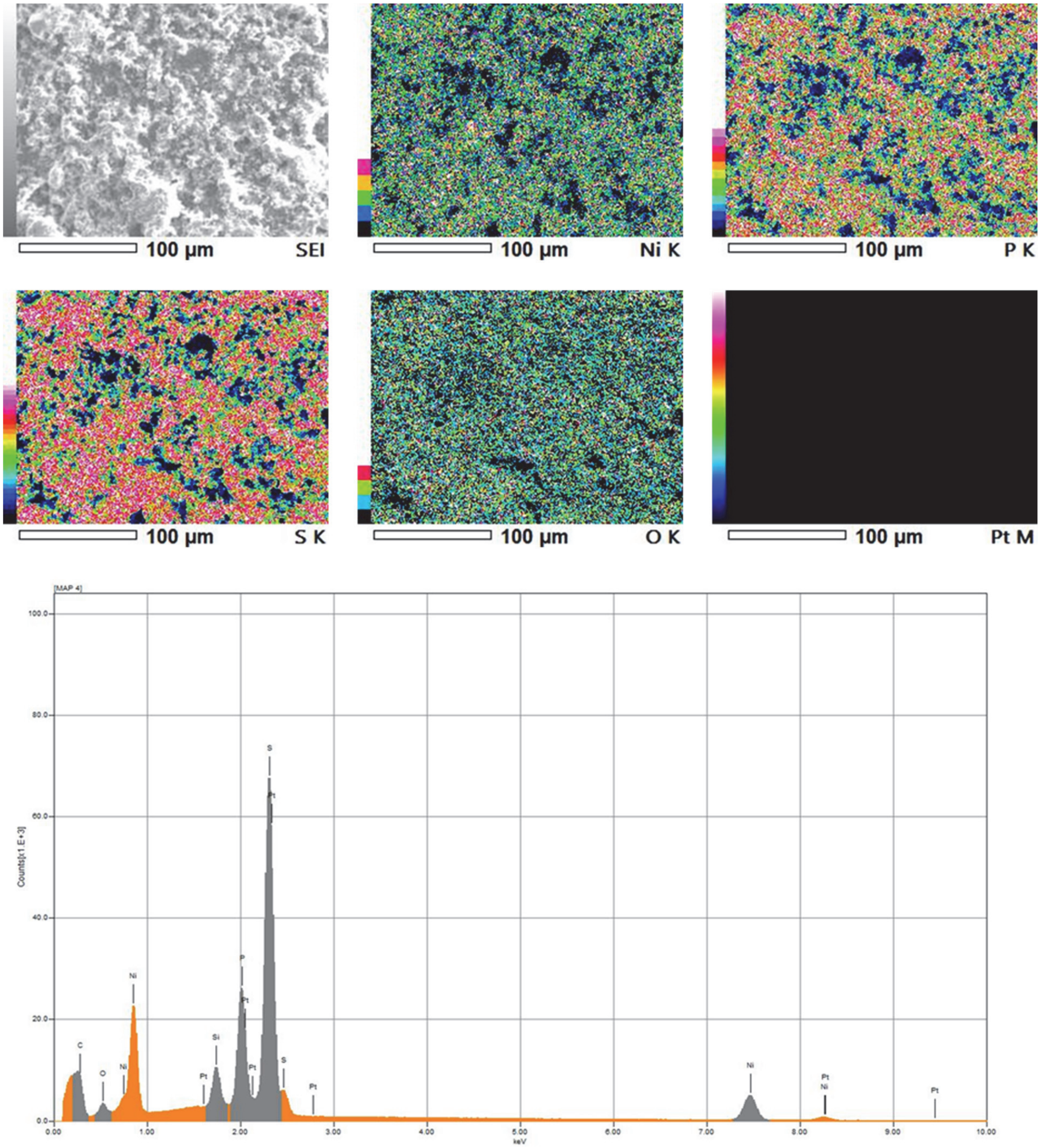

Figure S10A. Microprobe elemental mapping and semiquantitative surface analysis of $NiPS_3$ particles embedded on C_{wax} tips after 18-hour CA experiments using a platinum CE. The colors in each microprobe image reflect relative intensity of that surface detected element. To the left of each graph is its legend of increasing elemental content represented by different colors: pink>red>orange>yellow>green>blue>black.

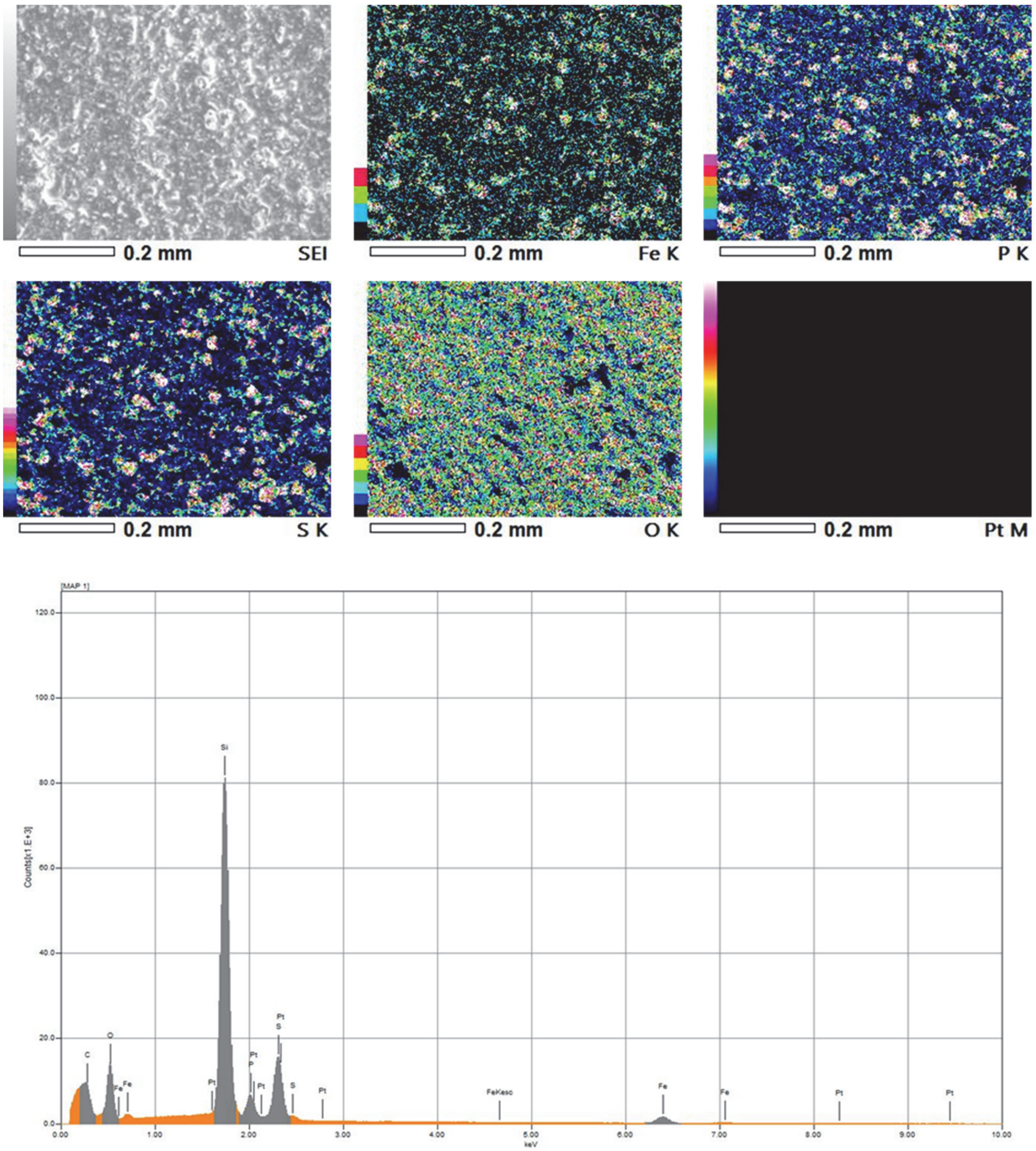

Figure S10B. Microprobe elemental mapping and semiquantitative surface analysis of $FePS_3$ particles embedded on C_{wax} tips after 18-hour CA experiments using a platinum CE. The colors in each microprobe image reflect relative intensity of that surface detected element. To the left of each graph is its legend of increasing elemental content represented by different colors: pink>red>orange>yellow>green>blue>black.

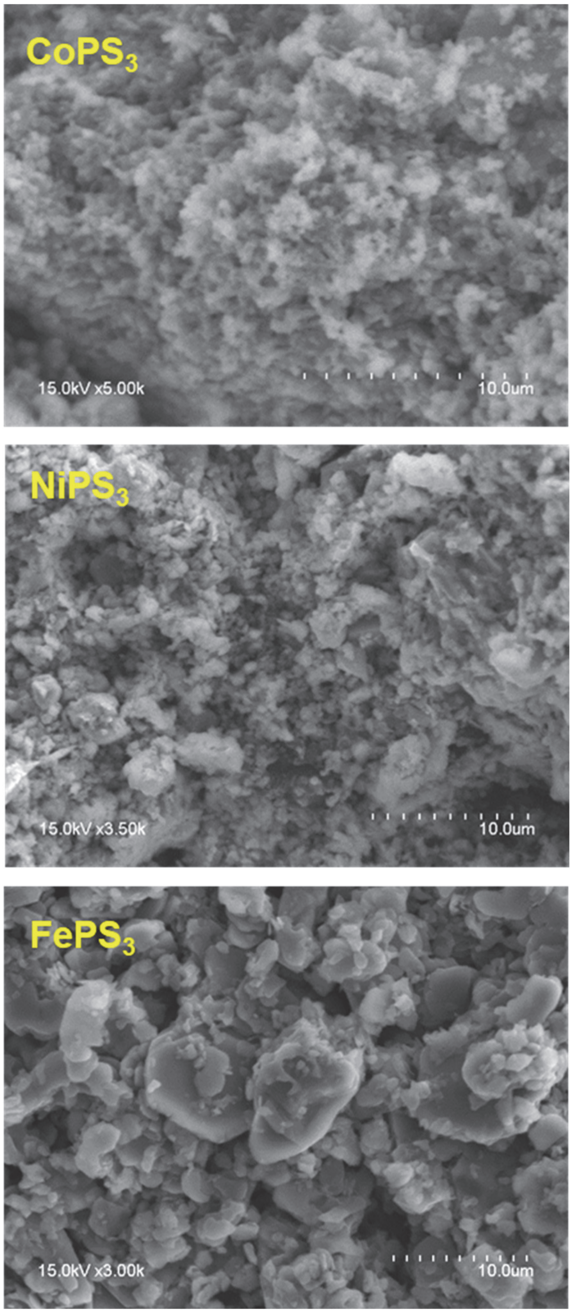

Figure S11. SEM images of MPS_3 materials embedded on the C_{wax} electrode surface after 18-hour HER CA experiment in 0.5 M H_2SO_4.

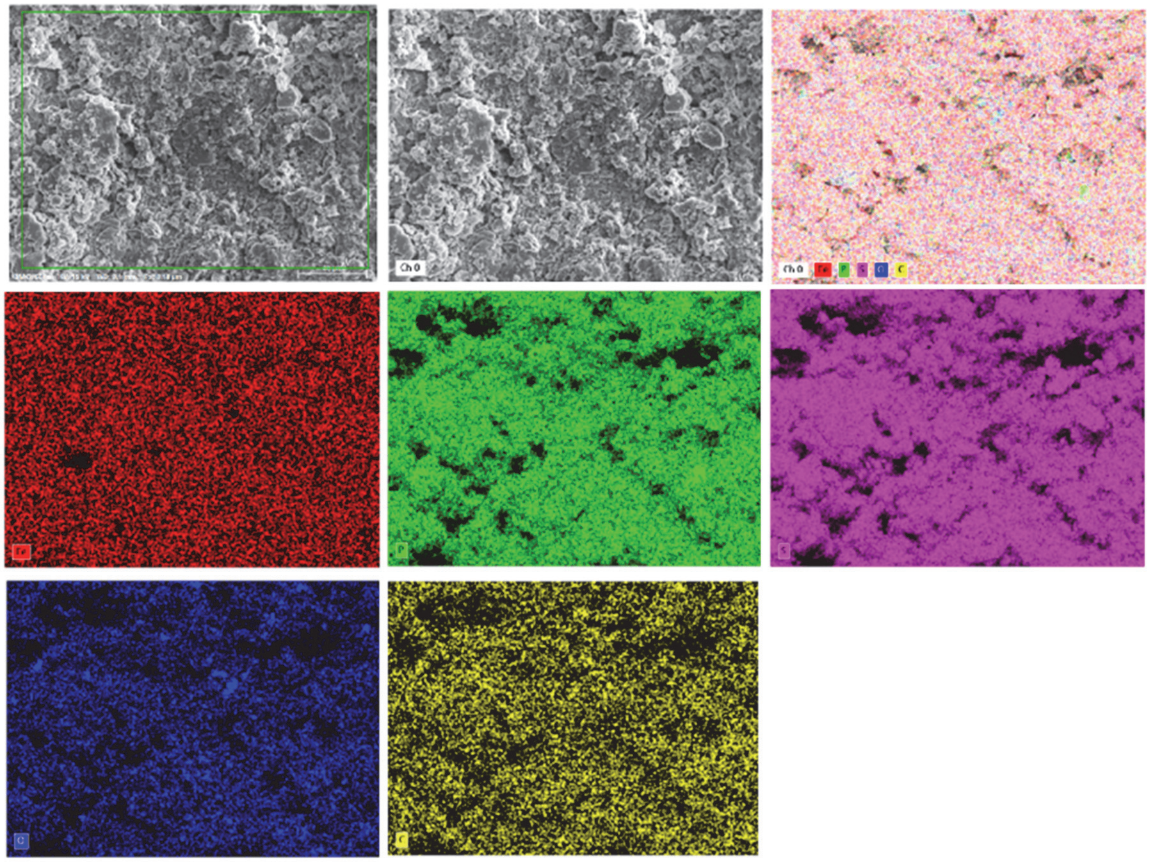

Figure S12A. EDS elemental mapping of $FePS_3$ particles embedded on C_{wax} tips after 18-hour CA experiments using a graphite CE. Semiquantitative elemental analysis gives Fe/P/S = 1/1.1/2.8. Scale bar in top left SEM image is 40 μm long.

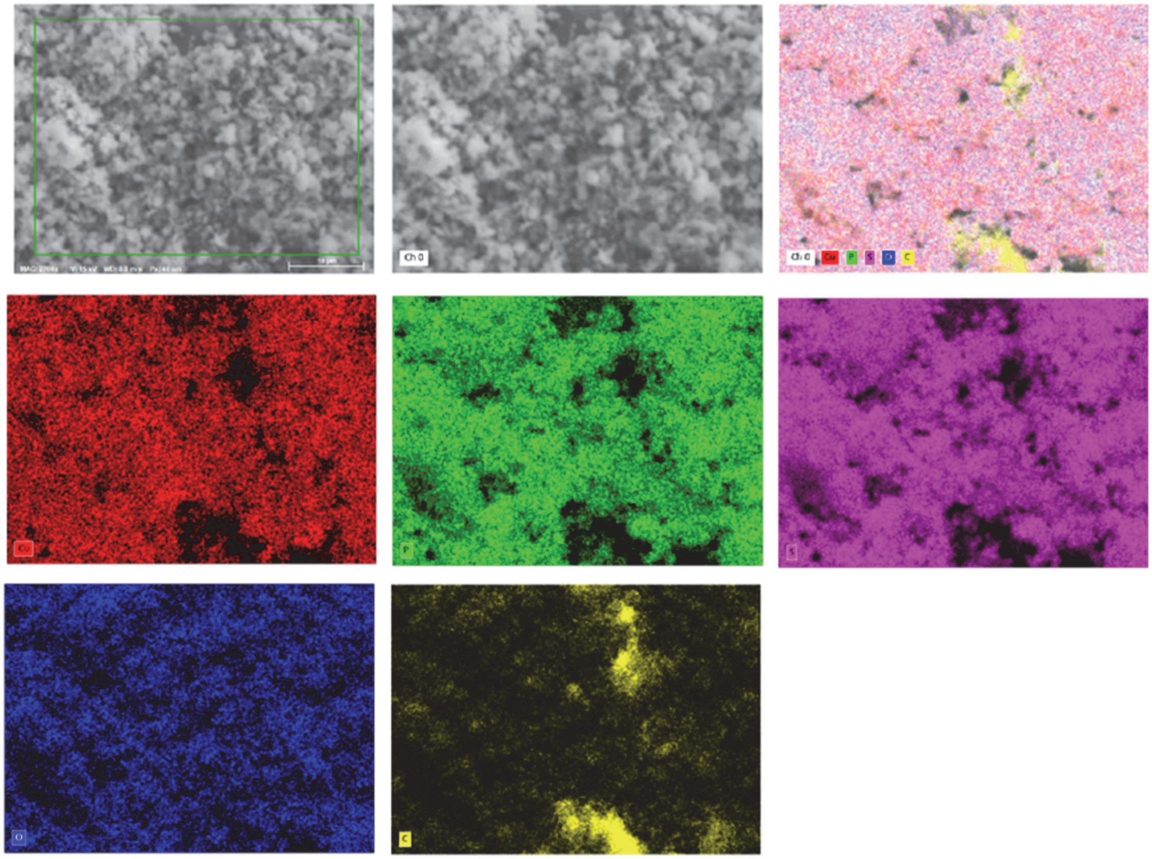

Figure S12B. EDS elemental mapping of $CoPS_3$ particles embedded on C_{wax} tips after 18-hour CA experiments using a graphite CE. Semiquantitative elemental analysis gives Co/P/S = 1/1.2/3.3. Scale bar in top left SEM image is 10 µm long.

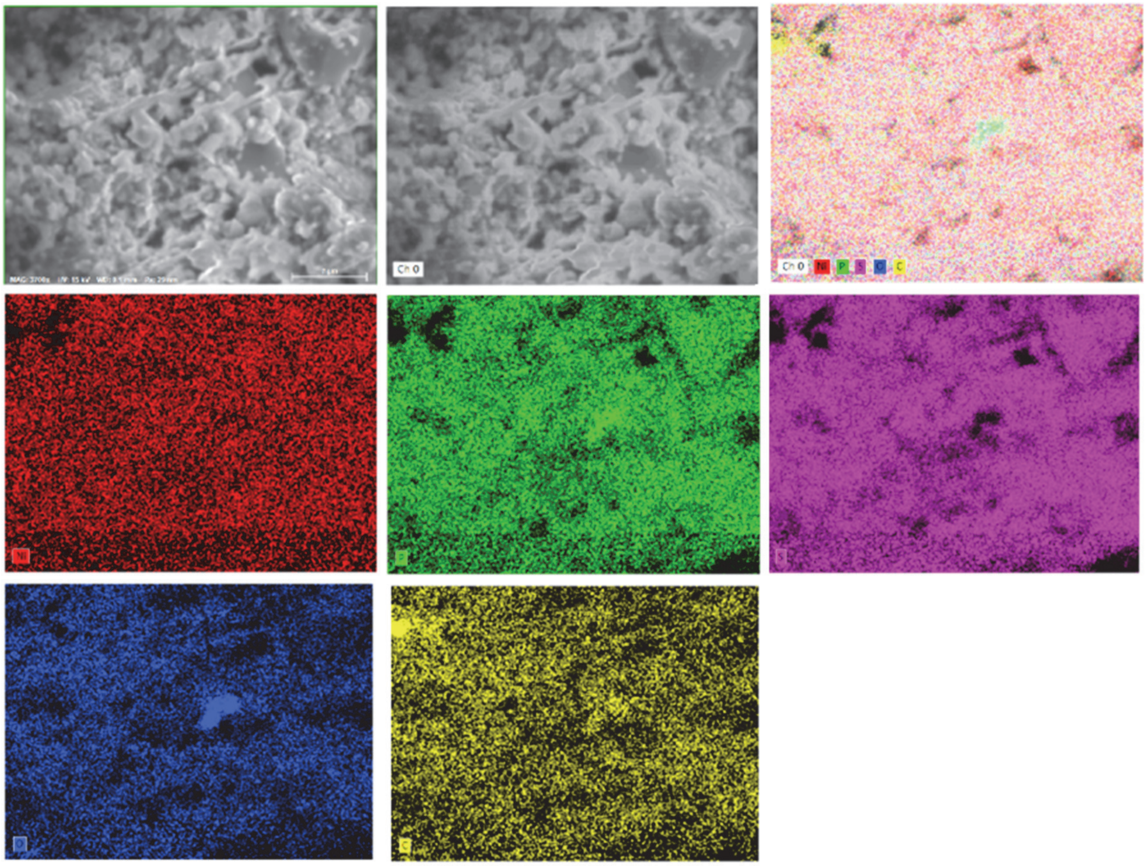

Figure S12C. EDS elemental mapping of $NiPS_3$ particles embedded on C_{wax} tips after 18-hour CA experiments using a graphite CE. Semiquantitative elemental analysis gives Ni/P/S = 1/1/2.6. Scale bar in top left SEM image is 7 µm long.

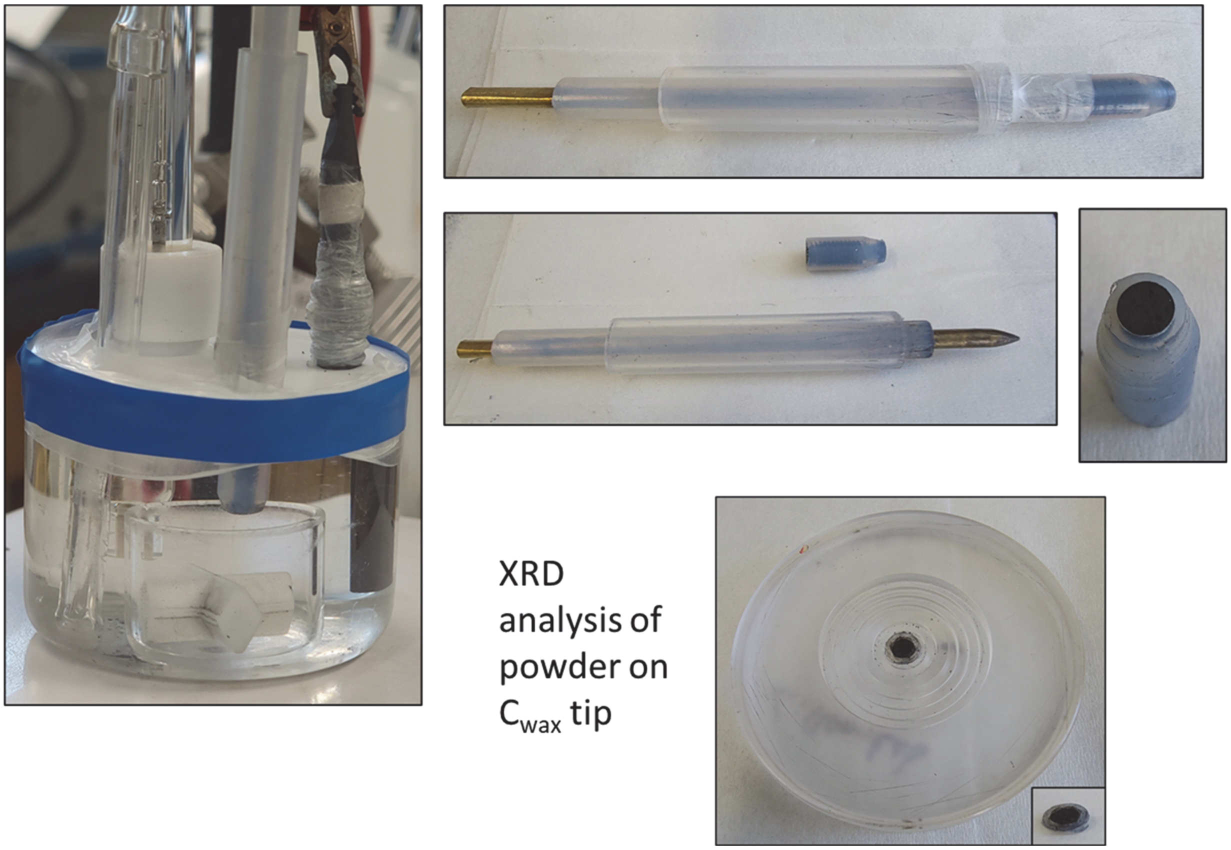

Figure S13. Electrochemical cell setup (top left) and images of C_{wax} electrode assembly and example of cut tips used for XRD analysis.

References

1. Ouvrard, G.; Brec, R.; Rouxel, J., Structural determination of some MPS3 layered phases (M = Mn, Fe, Co, Ni and Cd). *Materials Research Bulletin* **1985,** *20* (10), 1181-1189.
2. Taylor, B. E.; Steger, J.; Wold, A., Preparation and properties of some transition metal phosphorus trisulfide compounds. *Journal of Solid State Chemistry* **1973,** *7* (4), 461-467.
3. Marzik, J. V.; Hsieh, A. K.; Dwight, K.; Wold, A., Photoelectronic properties of Cu3PS4 and Cu3PS3Se single crystals. *Journal of Solid State Chemistry* **1983,** *49* (1), 43-50.
4. Brec, R.; Schleich, D. M.; Ouvrard, G.; Louisy, A.; Rouxel, J., Physical properties of lithium intercalation compounds of the layered transition-metal chalcogenophosphites. *Inorganic Chemistry* **1979,** *18* (7), 1814-1818.
5. Brec, R.; Ouvrard, G.; Louisy, A.; Rouxel, J., The Influence, on Lithium Electrochemical Intercalation, of Bond Ionicity in Layered Chalcogenophosphates of Transition-Metals. *Solid State Ionics* **1982,** *6* (2), 185-190.
6. Fuentealba, P.; Cortes, C.; Audebrand, N.; Le Fur, E.; Paredes-Garcia, V.; Venegas-Yazigi, D.; Manzur, J.; Spodine, E., First copper(ii) phase M'0.2Mn0.8PS3.0.25H2O and analogous M' = Co(II), Ni(II) and Zn(II) materials obtained by microwave assisted synthesis. *Dalton Trans* **2015,** *44* (28), 12493-6.
7. Itthibenchapong, V.; Kokenyesi, R. S.; Ritenour, A. J.; Zakharov, L. N.; Boettcher, S. W.; Wager, J. F.; Keszler, D. A., Earth-abundant Cu-based chalcogenide semiconductors as photovoltaic absorbers. *Journal of Materials Chemistry C* **2013,** *1* (4), 657-662.
8. Brec, R., Review on Structural and Chemical-Properties of Transition-Metal Phosphorus Trisulfides Mps3. *Solid State Ionics* **1986,** *22* (1), 3-30.
9. Barry, B. M.; Gillan, E. G., A General and Flexible Synthesis of Transition-Metal Polyphosphides via PCl3 Elimination. *Chemistry of Materials* **2009,** *21* (19), 4454-4461.
10. Coleman, N., Jr.; Lovander, M. D.; Leddy, J.; Gillan, E. G., Phosphorus-Rich Metal Phosphides: Direct and Tin Flux-Assisted Synthesis and Evaluation as Hydrogen Evolution Electrocatalysts. *Inorg Chem* **2019,** *58* (8), 5013-5024.
